# Milk Replacers and Bovine Spongiform Encephalopathy in Calves, Japan

**DOI:** 10.3201/eid1403.070852

**Published:** 2008-03

**Authors:** Toshiyuki Tsutsui, Takehisa Yamamoto, Sayaka Hashimoto, Takashi Nonaka, Akiko Nishiguchi, Sota Kobayashi

**Affiliations:** * National Institute of Animal Health, Ibaraki, Japan; † Headquarters, Fertilizer and Feed Inspection Services, Saitama, Japan

**Keywords:** BSE, case-control study, milk replacer, tallow, letter

## Abstract

Milk Replacers and Bovine Spongiform Encephalopathy in Calves, Japan

**To the Editor:** Milk replacers produced from a specific feed factory in Japan were suspected of being associated with a cluster of bovine spongiform encephalopathy (BSE) infection in calves. We conducted a case–control study to test this association.

In Japan, BSE infection has been confirmed in 32 calves as of the end of May 2007; 13 of these calves were born between December 1995 and August 1996. One BSE-infected calf was born in 1992 and had an atypical BSE phenotype (*1*). Because no BSE-infected calves were born in 1997 and 1998, we considered that those born in 1995 and 1996 formed an independent temporal cluster (Figure). Epidemiologic investigation showed that all 13 calves were fed milk replacers produced by a specific factory. Ten calves were born in Hokkaido, and 3 were born in the Kanto region, which is ≈800 km away from Hokkaido.

**Figure:**
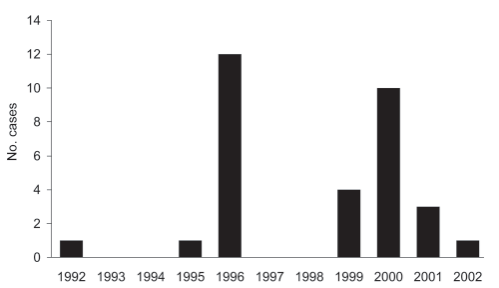
Number of cases of bovine spongiform encephalopathy by calves’ birth year, Japan.

In the case–control study, all farms where the 13 BSE-infected calves were born and raised for at least 1 year were defined as case farms. Control farms were defined as dairy farms where no BSE calves had been reported. Candidates for control farms comprised 200 randomly selected farms, which were located in 23 prefectures where the milk replacers were distributed. We used a national cattle identification database for random selection. Veterinary officers from the local government interviewed farmers in November and December 2006 and requested that they complete a questionnaire on farming practices in 1996, including herd size and use of milk replacers and calf concentrates. For the case farms, information previously obtained from outbreaks was used. Of the 200 potential control farms, 154 farms were used as controls. Forty-six farms were excluded; 24 farmers did not respond or could not specify the use of milk replacers; and 22 farms had either closed or farmers did not respond for miscellaneous reasons.

Among the 154 control farms, 36 farms (23%) used the milk replacers from the specific factory, 89 farms (58%) used other milk replacers, and 29 farms (19%) did not use milk replacers. Since 1 case farm lacked documented evidence about the use of the specific milk replacers, we conservatively assumed that 12 of 13 case farms used the specific milk replacers. We estimated the odds ratio for this risk factor by using logistic regression analysis. Our results indicated that the use of the milk replacers produced by the specific factory was associated with BSE infection (odds ratio [OR] 39.3, 95% confidence interval [CI] 4.9–312.9, p = 0.0005).

The milk replacers produced by the specific factory contained tallow that was produced at domestic rendering factories and imported from the Netherlands. Milk replacers were fed to calves during a relatively short period after birth (an average of 79 and 68 days, for case and control farms, respectively). If 1 production lot of milk replacer became accidentally contaminated with BSE, the exposure would occur in newborn calves within a relatively short period. This contamination may explain why 11 of 13 BSE-infected calves were born within a 2-month period from February 10, 1996, to April 8, 1996.

In Hokkaido, 9 of 10 BSE-infected calves were fed calf concentrates produced in the same feed factory. This proportion was higher than that of the 50 control farms in Hokkaido (22/50, Fisher exact test, p = 0.013). The calf concentrates might have become contaminated with meat-and-bone meal (MBM) because this factory used MBM for other animal feed. Multivariate logistic regression analysis, including this factor and that for the specific milk replacers, did not indicate significant association between the specific calf concentrates and occurrence of BSE calf concentrates: OR 3.2 (CI 0.8–13.0), p = 0.14; milk replacers: OR 21.7 (CI 2.5–192.6), p = 0.006. The factory that provided the specific concentrates belonged to a company affiliated with the company that produced the milk replacers in question. Given the fact that farmers tend to use milk replacers and calf concentrates from the same company, association of the calf concentrates with the BSE infection may have been masked by the use of specific milk replacers. However, our study is limited by the small number of BSE cases and investigation of events that occurred 10 years ago.

A possible causal association between the feeding of potentially contaminated milk replacers to calves and the occurrence of BSE has been suggested by several epidemiologic studies (*2**–**5*). However, no report shows experimental transmission of BSE by use of tallow or milk replacers (*6*). This lack of evidence in the literature may suggest that the risk of contracting BSE from processed tallow or milk replacers is low (*7*). If MBM is excluded as a source of infection, other transmission mechanisms, such as the feeding of animal fat, may become more important.

 This research was conducted under a research project for using advanced technologies in agriculture, forestry, and fisheries.
